# A rapid risk analysis tool to prioritise response to infectious disease outbreaks

**DOI:** 10.1136/bmjgh-2020-002327

**Published:** 2020-06-07

**Authors:** Dyah A S Lesmanawati, Patrick Veenstra, Aye Moa, Dillon C Adam, Chandini Raina MacIntyre

**Affiliations:** 1 Center for Tropical Medicine, Universitas Gadjah Mada, Yogyakarta, Daerah Istimewa Yogyakart, Indonesia; 2 District Health Office, Yogyakarta City, Yogyakarta, Indonesia; 3 Department of Infomatics, King's College London, London, UK; 4 Biosecurity Program, The Kirby Institute, Kensington, New South Wales, Australia; 5 Arizona State University College of Health Solutions, Phoenix, Arizona, USA

**Keywords:** public health

## Abstract

Epidemics are influenced by both disease and societal factors and can grow exponentially over short time periods. Epidemic risk analysis can help in rapidly predicting potentially serious outcomes and flagging the need for rapid response. We developed a multifactorial risk analysis tool ‘EpiRisk’ to provide rapid insight into the potential severity of emerging epidemics by combining disease-related parameters and country-related risk parameters. An initial set of 18 disease and country-related risk parameters was reduced to 14 following qualitative discussions and the removal of highly correlated parameters by a correlation and clustering analysis. Of the remaining parameters, three risk levels were assigned ranging from low (1) moderate (2) and high (3). The total risk score for an outbreak of a given disease in a particular country is calculated by summing these 14 risk scores, and this sum is subsequently classified into one of four risk categories: low risk (<21), moderate risk (21–29), high risk (30–37) and extreme risk (>37). Total risk scores were calculated for nine retrospective outbreaks demonstrating an association with the actual impact of those outbreaks. We also evaluated to what extent the risk scores correlate with the number of cases and deaths in 61 additional outbreaks between 2002 and 2018, demonstrating positive associations with outbreak severity as measured by the number of deaths. Using EpiRisk, timely intervention can be implemented by predicting the risk of emerging outbreaks in real time, which may help government and public health professionals prevent catastrophic epidemic outcomes.

**Key questions**

**What is already known?**

- There has been an increase in the number of serious infectious disease outbreaks worldwide in recent years.
- Rapid response time is critical for epidemic-prone diseases, and short delays to outbreak response result in preventable morbidity and mortality.
- Risk analysis can be used to prioritise a response to an epidemic by examining the potential impact of the infectious disease outbreak.

**What are the new findings?**

- EpiRisk provides rapid risk prediction of outbreaks that will assist decision makers in epidemic management.
- Both pathogen and country parameters have a significant impact on the risk of infectious disease outbreaks in particular countries or regions.
- EpiRisk provides an individualised approach where specific input data for a country and disease can be used, rather than a standard ‘one size fits all’ approach that would be less generalisable.

**What do the new findings imply?**

- The development of a simple risk analysis tool will be useful for global epidemic control. This tool can be used to rapidly predict the risk of outbreaks, which is useful when planning and prioritising interventions or for epidemic preparedness.
- An appropriate and timely intervention can help governments and public health professionals prevent catastrophic outcomes.

## Introduction

Since 2000, there has been an escalation in the number of serious infectious disease outbreaks worldwide.^1 2^ For example, the 2002–2003 severe acute respiratory syndrome coronavirus (SARS-CoV) epidemic spread to 37 countries, resulting in over 8000 cases and more than 700 deaths.^3^ Five years later in 2009, a new influenza subtype, A/H1N1pdm09 emerged in North America before circulating around the world affecting an estimated 24% of the global population.^4 5^ The 2013–2016 Ebola outbreak in West Africa was an unprecedented catastrophe, and Ebola continues to pose a global threat.^6^ Zika virus spread from Brazil and infected an estimated 1.3 million people, resulting in upwards of 4000 cases of microcephaly among infants.^7 8^ Outbreaks of acute flaccid myelitis have also occurred across the USA, Europe and Asia, associated with EV-D68.^9^ There has also been an increase in the number of vaccine-preventable disease outbreaks in recent years including polio,^10^ measles^11^ and diphtheria^12^.

Lessons learnt from recent serious epidemics show that control measures may not be sufficient or timely enough,^13^ and even short delays to outbreak response result in preventable morbidity and mortality.^3 14 15^ The response to the 2013–2014 West African Ebola outbreak is an example of a delayed intervention. Traditional laboratory and surveillance systems misdiagnosed Ebola victims as Lassa fever and cholera.^15^ The first confirmed laboratory case detected by scientists in France occurred approximately 2.5 months after illness in the index case, and intervention by WHO was delayed for at least 5 months after that.^15 16^ This failure in the early stages of the outbreak had significant follow-on effects, contributing substantially to an exponential increase in epidemic size to over 28 000 cases and 11 000 deaths.^6^ In 2015, 21 polio-free countries reported re-emergence of the virus with a total of over 1000 confirmed cases,^10^ with a slow response implicated as a factor in the impact of these outbreaks.^17^ Rapid response time is critical for epidemic-prone diseases, and decision support tools to prompt rapid response may be useful.

When looking at global patterns, there is diversity in the severity and impact of outbreaks,^18^ which means in the early stages, tools that can predict risk may help identify those with catastrophic potential. Both pathogen and country parameters have a significant impact on the risk of infectious disease outbreaks in a particular country or region.^19^ For example, innate characteristics of the pathogen such as the type of pathogen, transmission mode, basic reproductive number, case fatality rate, as well as the availability of effective therapy and vaccine for that particular disease all have a role in predicting the risk of an epidemic.^20^ Country factors including the social, economic and cultural characteristics of a country may also contribute to the risk of an outbreak. Risk assessment frameworks exist for polio, measles and dengue, which are used in endemic countries such as the Philippines and Romania.^21–23^ The availability, however, of a simple, universal epidemic risk scoring framework is valuable to predict the risk of an infectious disease outbreak and to prioritise response.

There are limited studies assessing the risk of infectious disease outbreaks based on country features. In 2017, Ajisegiri *et al* 19 used such an approach to predict the risk and outcome of the Ebola epidemic worldwide by considering country-specific parameters based on socioeconomic, health system, cultural and geographical factors. The risk framework by Ajisegiri successfully stratified the risk of Ebola outbreaks by country and predicted a much higher risk in West Africa compared with the USA or UK. Had such a tool been available in March 2014, it may have helped prompt a more rapid response. This study aimed to develop a generalised epidemic risk analysis framework that incorporates both pathogen and country-specific parameters to classify the risk of epidemics and provides an early warning system for the management of global outbreaks.

## Methods

We developed a risk analysis framework to predict the risk of an epidemic of any cause modifying the approach used in the Ebola-specific tool developed by Ajisegiri *et al*. In 2018, Ajisegiri *et al* 19 identified the role of sociodemographic features of the Ebola-affected countries to the magnitude of the outbreak. The authors developed a framework that assigned risk scores (from 1 to 3) to country-specific characteristics such as socioeconomic, health systems and geographical factors, as well as cultural beliefs and traditional practices. These risk scores were then added into a simple summation model to produce a single risk figure for a given country. Our modified approach differs from that by Ajisegiri *et al* in two key ways. First, we expanded the scope of the framework developed by Ajisegiri *et al* 19 to be suitable for almost any outbreak in any country (pending data availability) by including both disease-specific and country-specific parameters. Second, we used a subset of the country parameters used by Ajisegiri *et al*. A key objective of the framework presented in this paper is to allow for a quick and simple evaluation of outbreak severity risk in countries around the world. Some factors used by Ajisegiri *et al* require data that are not easily or quickly available (eg, screening at borders and bush meat consumption), and while those factors were suitable for evaluating a local region such as that in the Ebola outbreak, it does not meet the needs of our global framework. Data were collected on outbreak parameters that fall into two different categories: disease-related parameters and country-related parameters, as outlined in table 1.

**Table 1:** Risk analysis framework scoring criteria

No	Risk factors	Risk score			Explanation
		1	2	3	
1	Disease identification	Clinical syndrome is diagnostic.	A simple laboratory test is diagnostic.	Advance or prolonged investigation is required for confirmatory diagnosis.	The diagnostic capacity to identify a disease is a crucial element of epidemic control. It depends on how complicated the diagnosis of disease is.^46^ The easier the disease is to be identified, the faster it will be managed. A disease that needs an advanced or prolonged investigation for confirmation is likely to result in worse outcome.
2	**Pathogen**	Others	Bacterial	Viral	The type of pathogen is associated with disease spread. While there may be exceptions, in general, a viral pathogen is likely to cause more widespread epidemic because of higher R0 values.^35^
3.	Reservoir	Animal	Environmental	Human	Different types of the primary reservoir will affect the outcome of the infectious disease in the community.^47^ A disease where the primary host is human, with human-to-human transmission has higher epidemic potential than a disease that spread from animal to human.
4	**Basic reproductive number**	<1	1.0–2.0	>2	The basic reproductive number (R0) is the number of secondary infections produced by the index patient.^36^ The epidemic threshold is R=1, and for epidemic conditions to be present, R0 must be >1. The higher the R0, the more difficult an epidemic will be to control.
5	**Mode of transmission**	Vector borne or other animal borne	Foodborne, waterborne and direct contact	Airborne or droplet	Different modes of transmission will affect the spread of infectious disease in the community.^36^ Diseases that spread via the respiratory route (airborne/droplet) are more likely to cause a widespread outbreak than a disease that spreads through animals or vectors.
6	**Asymptomatic transmission**	No		Yes	A disease with transmission during the asymptomatic phase has more potential to cause a severe epidemic, because transmission from an asymptomatic patient is likely to be undetected.^24^
7	**Case fatality rate**	<1%	1.0%–5.0%	>5%	Potential mortality impact of the disease can be used as an indicator in risk prediction.^48^
8	**Therapy/drug availability**	Yes		No	The spread of disease will be difficult to control if there is no definitive therapy available for the disease. With definitive therapy, the spread of the disease may be minimised by reducing the natural duration of the disease, infectious period and the severity of the disease outcomes.^38^
9	**Vaccine availability**	Yes		No	Implementation of a vaccination programme is an effective control measure to reduce the risk of disease outbreaks.^37^ Vaccine availability through the Expanded Immunisation Program (EPI) indicates that the vaccine will be easily procured and accessible. Other vaccines may not be as accessible, even if recommended on a National Immunisation Program, depending on the country. Some vaccines are experimental and only available in trial conditions.
10	**Income**	High-income countries (>$12 056)	Middle-income countries ($995–$12 056)	Low-income countries (<$995)	The country’s resources and capacity for outbreak control are associated with the income.^49^ The World Bank divides countries into four categories—high, middle (upper and lower) and low income—based on world income percentile.
11	**HE total (% of GDP**)	Upper third (>10.0%)	Middle third (5.0%–10.0%)	Lower third (<5.0%)	Health expenditure (HE) indicates the proportion of country GDP that is assigned to the health sector expenditure. The proportion of health expenditure in a country has a strong association with the healthcare services provided to the population.^50^ A country’s health expenditure was divided into upper, middle and lower third.
12	**Peace index**	High peace (<2.0)	Middle peace (2.0–2.3)	Low peace (>2.3)	The state of peace may impact the country’s health system. In a conflict-affected country, access to essential services is poor. As a consequence, the population in that country become more vulnerable to infectious disease transmission. In addition, detection and control of the outbreak is a challenge in the conflict-affected populations. The Institute for Economics and Peace divided state of peace into five different groups: very high, high, medium, low and very low.^51^ However, for this framework, we categorised the state of peace into three groups: high (combination of very high and high group), medium and low peace (combination of very low and low).
13	**Land border**	Maritime only (island nation)	Mixed maritime–land	Land only	The country border is associated with the mobility of people, accessibility of transportation modes as well as the time required to travel.^52^ A land border has a higher likelihood of interborder disease transmission than water border because of easier access.
14	Transport network	<2	2–4.5	>4.5	The transportation network is an important determinant of health. It affects mobility from one area to another. In an outbreak event, the transportation network has a major role in the possible disease transmission. The better transportation network that the country has also increases the likelihood of disease transmission. The World Economic Forum measures the quality of transportation infrastructure based on a country’s roads, railways and air transport infrastructure data.^53^ We combined the country’s score of roads, railways and air transport infrastructure and divided them into three different categories of low, medium and high.
15	**Population density**	<100/km^2^	100–1000/km^2^	>1000/km^2^	Overcrowding is one of the major factors in the disease transmission risk.^31^ Epidemics of disease may be more severe in high-density populations than low-density populations.
16	**Physician density**	>2.9/1000 populations	0.8–2.9/1000 populations	<0.8/1000 populations	Adequate availability and skill of physicians are critical for a country to attain population health goals through the provision of sufficient basic medical care. Lack of availability and accessibility to the physician service could aggravate the impact of an outbreak because of delay in medical treatment. WHO report^54^ indicates the range of physician density per 1000 population; <0.8 as low, 0.8–2.9 as middle and >2.9 as high.
17	Nurses and midwife density	>7.1/1000 populations	1.7–7.1/1000 populations	<1.7/1000 populations	Nurses and midwives play a crucial role in healthcare services, both in the hospital and community settings. Sufficient number and capacity of nurses and midwives would be associated with outbreak impact by reducing potential disease transmission in the population. WHO report^54^ categorises population-weighted density of nurse and midwife in three groups: low, moderate and high.
18	**Hospital beds**	>4 beds/1000 populations	>2–4 beds/1000 populations	0.1–2 beds/1000 populations	Hospital beds are an indicator of available resources to deliver healthcare services. Without sufficient hospital beds during an outbreak event, the likelihood of uncontrolled transmission increases.^21^ When insufficient resources force patients to stay longer in the community, the risk of disease transmission in the community increases.

Factors in bold were included in the final EpiRisk tool after qualitative analysis and a correlation analysis.

GDP, gross domestic product.

### Selection of initial risk parameters and data collection

We searched the literature to identify common risk factors of infectious disease outbreaks using three online databases: PubMed/Medline, Scopus and CINAHL using keywords ‘outbreak* OR epidemic* OR pandemic* OR emerging disease* OR re-emerged disease*’. We identified numerous initial factors associated with the severity of the disease outbreaks,^24–27^ which we categorised as either disease-related parameters or country-related parameters. Additional disease-related parameters were selected based on a modified Disease Attribute Intelligence System risk assessment tool by the Institute of Environmental Science and Research Limited,^20^ while additional country-related parameters were identified based on the risk analysis framework tool by Ajisegiri *et al*.^19^ The financial capability and the level of investment of a country plays a significant role in the early response to outbreaks.^28^ Sociopolitical factors also contribute to the length and outcome of outbreaks. For example, war and conflict provide ideal conditions for outbreaks of infection diseases. In conflict areas, health professionals flee, infrastructure is destroyed and the supply of medical equipment is halted.^29^ In some instances, hampering of immunisation efforts has also contributed to the spread of vaccine-preventable diseases,^30^ making a country more vulnerable to outbreaks. The majority of severe disease outbreaks also originate from densely populated regions.^31^ Overcrowding coupled with lower living standards can lead to the efficient transmission of diseases and positively correlate with the risk of an outbreak.^32^ The existing health system within the country also plays a crucial role in the risk of outbreaks. An epidemic can spread wider and faster in a country with a weak health system.^33^

The infectiousness of pathogens also contribute to the risk level of an outbreak.^34^ Diseases caused by viral pathogens are likely to transmit more rapidly due to the nature of viral replication and mode of transmission in contrast to many bacteria;^35^ pathogens such as measles are more infectious partly because they spread by the respiratory route and have a high R0.^36^ Cholera, although spread via the faecal–oral route, is not as rapid as airborne transmission; however, it has a high reproductive number.^36^ During the South Sudan epidemic in 2016 cholera spread rapidly, resulting in over 20 000 cases and 436 deaths. Besides infectiousness, the availability of control measures for prevention and treatment also has an impact on the overall risk of an outbreak. These factors make it easier to prevent and control epidemics. Vaccination reduces the risk of infections by reducing the number of susceptible people in the population.^37^ Treatment availability affects the risk of disease outbreak by reducing the duration, infectious period and severity of disease.^38^

Initially, 18 parameters were selected to calculate overall epidemic risk (table 1). Explanations of each parameter can also be seen in table 1. Sources of data for country-related parameters included the World Bank, WHO Global Health Observatory and the Peace Institute. For disease-related parameters, a mixture of grey literature and official sources including Centers for Disease Control and Prevention and WHO were used.

The preparation of country-specific data involved collection of both current and historical data points for 198 countries from the World Bank database. The historical data were required to allow for a valid evaluation of the tool on historical outbreaks. That is, we used country-specific data points (eg, gross domestic product and physicians’ numbers) that were relevant at the time of the outbreaks used for evaluation.

As part of the collection of pathogen-specific data, we performed a review of three outbreak databases: ProMED-mail,^39^ Healthmap^40^ and EpiWatch^41^ to collect a line-list of diseases from recent outbreak events for evaluation of the tool.

### Criteria for allocating risk scores

We applied values ranging from one to three for each parameter to indicate the level of risk, where ‘low risk’ was denoted by ‘1’, ‘moderate risk’ was denoted by ‘2’ and ‘high risk’ was denoted by ‘3’. We applied minimum and maximum values, score 1 as ‘yes’ and 3 as ‘no’ for binary parameters that had only two different risk groups, for example, asymptomatic transmission, vaccine availability and drug availability. Criteria for assigning risk scores to selected parameters were used from the relevant studies.^19 20^ The values applied were based on specific risk factor criteria and are detailed in table 1. Where there was no available data for a parameter (N/A=not available), the highest risk score was assigned.

### Selection of final parameters

The initial set of 18 parameters were reduced to 14 as a result of qualitative discussions and a correlation and clustering analysis on the country parameters. The country parameter ‘roadways/transport network’ was removed due to conflicting interpretations of risk i.e. (1) it may indicate better access to healthcare services, or (2) increase contact between people and hence risk of infection. The disease-factor ‘Disease identification method’ was removed as it provided little variance with most diseases requiring further investigation for confirmatory diagnosis. The ‘reservoir’ factor was removed as its mapping to risk is not consistent and straightforward.

**Figure 1:**
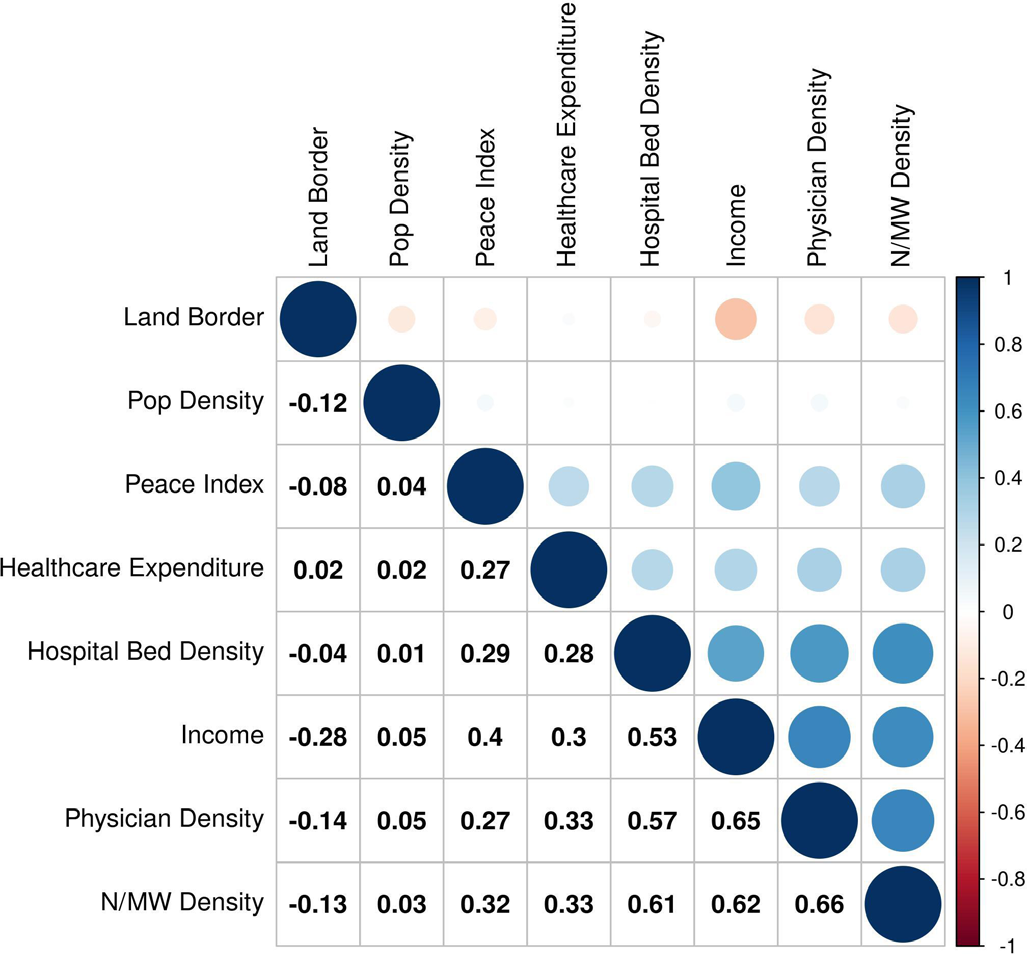
Kendall correlation coefficients between country factors. N/MW, nurses and midwife.

After the qualitative determination to remove the three aforementioned factors, leaving 15 factors (eight county, seven disease), we performed a quantitative analysis to compute the correlations between all remaining country parameters for the 198 countries in our dataset. As some of the country factors (see table 1) consist of ordinal level data, we used the Kendall correlation coefficient to compute the correlations. See figure 1 for the results between the eight country factors.

The correlation results demonstrated that income, hospital bed density, physician density, and nurses and midwife density were highly correlated factors. We decided that income and hospital bed density were both useful information to show in the framework, and we include them both, despite the Kendall correlation of 0.53. This will lead to a bias towards this in the model, which we address in our conclusion. Regarding the physician density and nurse/midwife density factors, their correlation is 0.66, and we decided they represent similar information about the healthcare system of a country; we therefore chose to retain physician density as a factor over nurse and midwife density, because of physician diagnostic and therapy skill and due to slightly more limited historical availability of nurse and midwife density data for some countries. The initial 18 parameters were reduced to 14 after the reduction process described above (the remaining factors are bold in table 1).

In our framework, an outbreak in a given country is given risk scores in each of the seven disease factors and seven country factors. These scores are then summed to produce a disease score and a country score for the outbreak, which are then in turn summed to produce a final risk score. The minimum possible risk score is 14 (all 1s) and the maximum is 42 (all 3s). The total score was classified into four risk categories to rank the priority. The categories are ‘low risk’ for a score less than 21, ‘moderate risk’ for a score between 21 and 29, ‘high risk’ for score 30–37 and ‘Extreme risk’ for total score beyond 37. The cut-offs were determined based on expert knowledge of historical outbreaks.

The historical and current country data, together with the disease data, are stored in csv files and managed in the R statistical software package for analysis. The initial data collection was managed in Excel.

### Patient and public involvement statement

Involvement of patients or public in this research was not applicable.

### Model evaluation

We evaluated our risk framework in two ways. First, to demonstrate the use of the framework, we applied it to a number of past outbreaks and showed that our computed risk scores provided insight into the severity of nine historic outbreaks. Second, we collected data on 61 different outbreaks between 2002 and 2018 and showed to what extent the risk scores computed in our model correlate with the number of cases and deaths in those 61 outbreaks.

First, to demonstrate the use of the framework, we selected nine different outbreaks with varied outcomes, from different countries with low, medium and high incomes from the last 5 years (2015–2019) in order to to cover a range of disease-related and country-related parameters. Table 2 presents these outbreaks together with the country and disease risk scores computed using our framework. The outbreaks consist of the following: hepatitis A in Australia (2018), Ebola in the USA (2018), measles in Japan (2018), diphtheria in Bangladesh (2017), Zika in Brazil (2015), hepatitis A in Italy (2013), Ebola in Sierra Leone (2014), cholera in South Sudan (2016) and Lassa fever in Nigeria (2018).

**Table 2:** Calibration of EpiRisk tool with nine historic outbreaks

	Hepatitis A (Australia)	Measles (Japan)	Hepatitis A (Italy)	Ebola (USA)	Zika (Brazil)	Diphtheria (Bangladesh)	Cholera (South Sudan)	Ebola (Sierra Leone)	Lassa fever (Nigeria)
Outbreak characteristics									
Duration	10 weeks	8 weeks	18 months	12 weeks	>10 months	6 weeks	18 months	19 months	13 months
Cases (suspected and confirmed)	30	161	1803	4	218 931	804	20 438	13 683	4466
Deaths (probable and confirmed)	1	0	1	1	11	15	436	3953	142
International aid received	No	No	No	No	Yes	Yes	Yes	Yes	Yes
Reference	55	56	57	58	59	60	59	61	62
Country parameters									
Income	1	1	1	1	2	2	3	3	2
HE total (% of GDP)	2	1	2	1	1	3	2	1	3
The state of peace	1	1	1	2	2	2	3	1	3
Country’s border	1	1	2	2	2	2	3	2	2
Physician density (per 1000)	1	2	1	2	2	3	3	3	3
Hospital bed density (per 1000)	2	1	2	2	2	3	3	3	3
Population density	1	3	3	1	1	3	1	2	3
Total country score	**9**	**10**	**12**	**11**	**12**	**18**	**18**	**15**	**19**
Disease parameters									
Pathogen	3	3	3	3	3	2	2	3	3
Basic reproductive number	2	3	2	3	3	3	3	3	3
Mode of transmission	2	3	2	2	1	3	2	2	2
Asymptomatic stage	3	3	3	1	3	3	3	1	3
Case fatality rate	1	3	1	3	3	3	3	3	3
Therapy/drug availability	3	3	3	3	3	1	1	3	3
Vaccine availability	1	1	1	3	3	1	1	3	3
Total disease score	**15**	**19**	**15**	**18**	**19**	**16**	**15**	**18**	**20**
Overall score	**24**	**29**	**27**	**29**	**31**	**34**	**33**	**33**	**39**
Risk classification	Moderate	Moderate	Moderate	Moderate	High	High	High	High	Extreme

These nine historical outbreaks were divided into three groups based on similarity of duration, cases/deaths and international aid:

Group 1: hepatitis A outbreak in Australia, Ebola outbreak in the USA and the measles outbreak in Japan. These three epidemics lasted for less than 3 months, had only a few cases, few fatalities and received no international support.

Group 2: diphtheria in Bangladesh, Zika virus in Brazil and hepatitis A in Italy all shared similar characteristics: the number of cases were high, but the case–fatality rate was low. There were 218 931 people affected with Zika outbreak in Brazil with only 11 deaths. The outbreak duration of this group varied from 6 weeks to 18 months. Both Bangladesh and Brazil obtained international aid, while Italy managed the outbreak with their own resources.

Group 3: Ebola in Sierra Leone, cholera in South Sudan and Lassa fever in Nigeria include large epidemics with a long duration and high case fatality and morbidity rates. All group 3 outbreaks required international aid to control the outbreak.

**Figure 2:**
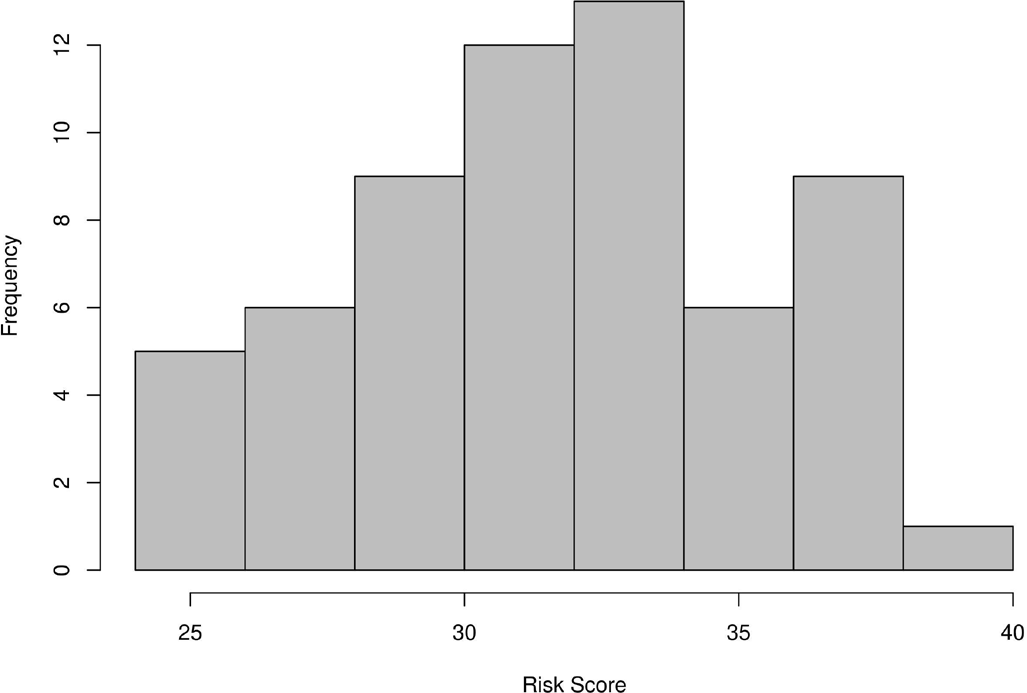
The distribution of risk scores computed with our risk framework for the 61 outbreaks in our evaluation dataset.

To demonstrate the extent to which the model risk scores correlate with outbreak severity, we collected data on 61 different (non-endemic) outbreaks between 2002 and 2018 of 18 different pathogens in 43 different countries with a wide range of number of cases (mean 25 691, median 804, first quantile 100, third quantile 2734) and deaths (mean 347, median 14, first quantile 1, third quantile 76) per outbreak. Figure 2 shows the distribution of the total risk score computed for the 61 outbreaks in the dataset.

For our evaluation, we split the outbreak risk scores into four quantiles, and we split the number of deaths into four quantiles. That is, Q1 of risk scores represents the 15 outbreaks in our evaluation set with the lowest risk scores and Q4 represents the 15 outbreaks in our evaluation set with the highest risk scores. Similarly, Q1 of deaths represents the 15 outbreaks that resulted in the lowest number of deaths and Q4 represents the 15 outbreaks that resulted in the highest number of deaths. We then tabulated these two quantile groups against each other to evaluate to what extent outbreaks with the highest number of deaths were also the outbreaks with the highest risk scores in our framework and similarly for the lower quantiles. See table 3 in the results section for the result.

**Table 3:** Tabulating the death quantiles of outbreaks against the risk score quantiles of the corresponding outbreaks

	Deaths				
Risk score		Q1	Q2	Q3	Q4
	Q1	7	7	0	1
	Q2	4	2	6	3
	Q3	1	2	4	8
	Q4	3	4	5	4

For example, the cell in the bottom right indicates that four of the outbreaks in the quantile with the highest death outbreaks also had top risk scores.

## Results

Table 1 presents the sourced parameters for each outbreak corresponding to the nine outbreaks listed in table 2 with the corresponding risk score and risk classification. The total risk scores in table 2 is the sum of the total country score and total disease score. Australia has the lowest country score, and the overall risk score for Australia is the lowest. Likewise, Japan had a low country score. In contrast, Nigeria had the highest total country score, while Bangladesh also had a high country score; however, diphtheria’s diseases-related score is the lowest among all diseases tested, so the overall risk score is not high enough to characterise the outbreak as extreme risk but was characterised as high-risk overall.

For our quantitative evaluation of risk scores computed on an evaluation dataset of 61 outbreaks, we first split the outbreaks into quantiles. Each of the 61 outbreaks was placed in one of four quantiles for the number of deaths associated with the outbreak. An outbreak in Q1 was an outbreak where the number of deaths was in the lowest 25% of the full dataset of 61 outbreaks. An outbreak in Q4 for the number of deaths has a number of deaths associated with it that is within the highest 25% of the full dataset. The same method is used to place the outbreaks into four quantiles of risk scores as computed by our risk framework. Table 3 shows the result. What is promising is that of the 16 outbreaks that resulted in the highest number of deaths (Q4), 12 were in the top 2 quantiles in terms of risk score (Q3/Q4). However, the other four were in the lower two quantiles of risk score. Of the 15 outbreaks in our dataset that resulted in the least number of deaths (Q1), 11 were in the bottom two quantiles in terms of risk (Q1/Q2) and four were in the top quantiles of risk (Q3/Q4). An equally promising pattern is shown for outbreaks in Q2 and Q3.

There is a clear association between the risk scores computed by our framework and the severity of outbreaks as measured by the number of deaths, but there are also outbreaks that fall outside of the risk categories one would wish to see. For example, one of the most severe outbreaks was in the bottom 25% of risk scores, and three of the least severe outbreaks were in the highest 25% of risk scores. Nonetheless, the overall association exists, and in future work, we intend to make the relationship stronger.

## Discussion

We found that risk calculations by EpiRisk correlated well with epidemic impact using our test dataset of historical epidemics. Both disease-related parameters and country-related parameters significantly contribute to the overall risk of epidemics. The EpiRisk tool is a simple and rapid risk scoring framework for global infectious disease outbreaks that could be used to prioritise rapid and effective epidemic response.^42^ Lessons from the severe acute respiratory syndrome (SARS) outbreak in early 2003 indicates that the timing of outbreak detection is a key factor in the success of interventions, and delays in outbreak detection can limit the efficacy of such interventions. Delays occurred in the 2013–2016 West African Ebola outbreak resulting in over 28 000 cases and over 11 000 deaths and had a substantial economic cost for the affected countries.^43^ The use of a risk scoring framework, if used early in the outbreaks, might have predicted the need for a rapid response and prevented a catastrophic outcome.^19^ This approach can be used if there are more than one epidemic within a country, particularly if resources are limited. An urgent and aggressive intervention is needed for a higher risk score. It includes aggressive case and contact identification, isolation and management and extreme social distancing nationally. Whereas a less aggressive intervention such as standard precaution and routine surveillance in the local level would be appropriate for a lower risk score. For example, an outbreak of Lassa fever occurring in Nigeria at the same time as other infectious diseases such as polio and cholera could also assist the government in prioritising urgent interventions to Lassa fever because it has higher risk disease and therefore minimise the overall impact. Such a tool may assist with prioritisation and early decision making. It may also help international organisations to prioritise countries in most need of urgent assistance.^28^

A limitation of this study is the dependence on the quality of the data inputs, such as the validity of the data sources, and the use of fixed disease parameters of known pathogens. The data used to calculate the risk scores were collected from various sources and from different points in time, which in some cases are out of date or contain biases. On an evaluation set of 61 outbreaks, our results show that the risk score computed using our framework has a positive association with outbreak severity as measured by number of deaths associated with an outbreak. In future work, we intend to improve the model to make this association stronger. At the moment, our model is a simple linear summation of risk factors. We intend to develop a larger dataset of historical outbreaks, at which point it will become viable to develop individual weights for the factors in the model that we hope will improve the strength of the association between risk score and outbreak severity. It should also be noted that the risk categories (and their cutoffs) do lead to a situation where a unit difference in risk can change the risk category entirely. The framework is also currently limited to known pathogens. This limits the tools application during the early moments of an outbreak where the pathogen maybe unknown and awaiting diagnostic confirmation. This also includes the emergence of novel pathogens, where many disease parameters required for input such as the basic reproductive number (R_0_), mode of transmission and case fatality rate are likely to be uncertain. However, in the case of COVID-19 as an example, as the pandemic progressed, these parameters quickly became known. Several of the high-scoring features outlined in table 1, such as asymptomatic transmission, respiratory spread, viral infection and high case fatality rate, were present.^44 45^ Future iterations of the tool could allow the input of these disease parameters manually, and various potential scenarios could be tested by varying the parameters individually, such are variations of R_0_ to account for uncertainty early in an epidemic. The framework is meant to be used as a whole, where the user ought to look at the risk score, the disease score, the country score and then the individual risk factor scores for deeper insight. Despite these limitations, EpiRisk provides an individualised approach where specific input data for a country and disease can be used, rather than a standard ‘one size fits all’ approach that would be even less generalisable. We believe this country-level approach is more rigorous and can predict outbreak risk more accurately than relying on disease or country factors in isolation.

The development of a simple risk analysis tool will be useful for global epidemic control. We have demonstrated in principle that EpiRisk can assess the level of epidemic risk for individual epidemics and performed well when tested against an initial set of real-world epidemics. This tool can be used to rapidly predict the risk of outbreaks that is useful when planning and prioritising interventions or for epidemic preparedness. An appropriate and timely intervention can help governments and public health professionals prevent catastrophic outcomes. To improve the relevancy for current or future prediction, the development of a real-time tool that provides the most current data for risk prediction is crucial. The involvement of local government and other health organisation to improve the data is also important. In future work, we will evaluate the generalisability of EpiRisk through the use of a more comprehensive test set of outbreaks.

## References

[1] Davey B Public health approaches to infectious disease, 2016.

[2] Bui CM, Chughtai AA, Adam DC, et al An overview of the epidemiology and emergence of influenza A infection in humans over time. Arch Public Health 2017;75:15. 10.1186/s13690-017-0182-z 28352464 PMC5366997

[3] Smith RD Responding to global infectious disease outbreaks: lessons from SARS on the role of risk perception, communication and management. Soc Sci Med 2006;63:3113–23. 10.1016/j.socscimed.2006.08.004 16978751 PMC7130909

[4] Van Kerkhove MD, Hirve S, Koukounari A, et al Estimating age-specific cumulative incidence for the 2009 influenza pandemic: a meta-analysis of A(H1N1)pdm09 serological studies from 19 countries. Influenza Other Respir Viruses 2013;7:872–86. 10.1111/irv.12074 23331969 PMC5781221

[5] World Health Organization Pandemic H1N1 2009: WHO regional office for south-east Asia, 2009.

[6] Coltart CEM, Lindsey B, Ghinai I, et al The Ebola outbreak, 2013-2016: old lessons for new epidemics. Philos Trans R Soc Lond B Biol Sci 2017;372:20160297. 10.1098/rstb.2016.0297 28396469 PMC5394636

[7] Roth A, Mercier A, Lepers C, et al Concurrent outbreaks of dengue, chikungunya and Zika virus infections - an unprecedented epidemic wave of mosquito-borne viruses in the Pacific 2012-2014. Euro Surveill 2014;19:20929. 10.2807/1560-7917.ES2014.19.41.20929 25345518

[8] WHO Epidemiological Neurological syndromes, congenital anomalies and Zika virus infection. Washington DC: PAHO/WHO, 2016.

[9] Dyda A, Stelzer-Braid S, Adam D, et al The association between acute flaccid myelitis (AFM) and enterovirus D68 (EV-D68) - what is the evidence for causation? Euro Surveill 2018;23. 10.2807/1560-7917.ES.2018.23.3.17-00310 PMC5792700 29386095

[10] Centers for Disease Control and Prevention (CDC) Resurgence of wild poliovirus type 1 transmission and consequences of importation--21 countries, 2002-2005. MMWR Morb Mortal Wkly Rep 2006;55:145. 16484977

[11] Filia A, Bella A, Del Manso M, et al Ongoing outbreak with well over 4,000 measles cases in Italy from January to end August 2017 - what is making elimination so difficult? Euro Surveill 2017;22. 10.2807/1560-7917.ES.2017.22.37.30614. [Epub ahead of print: 14 Sep 2017]. PMC5607657 28933342

[12] Harapan H, Anwar S, Dimiati H, et al Diphtheria outbreak in Indonesia, 2017: an outbreak of an ancient and vaccine-preventable disease in the third millennium. Clin Epidemiol Glob Health 2018.

[13] WHO Ebola Response Team, Aylward B, Barboza P, et al Ebola virus disease in West Africa--the first 9 months of the epidemic and forward projections. N Engl J Med 2014;371:1481–95. 10.1056/NEJMoa1411100 25244186 PMC4235004

[14] Hoffman SJ, Silverberg SL Delays in global disease outbreak responses: lessons from H1N1, Ebola, and Zika. Am J Public Health 2018;108:329–33. 10.2105/AJPH.2017.304245 29345996 PMC5803810

[15] World Health Organization Origins of the 2014 Ebola epidemic: one year into the Ebola epidemic, 2015.

[16] Hein W The response to the West African Ebola outbreak (2014-2016): a failure of global health governance?: international health law: lessons from the Ebola crisis and beyond, 2017.

[17] Akil L, Ahmad HA The recent outbreaks and reemergence of poliovirus in war and conflict-affected areas. Int J Infect Dis 2016;49:40–6. 10.1016/j.ijid.2016.05.025 27237735 PMC4975965

[18] Abdullah ASM, Tomlinson B, Cockram CS, et al Lessons from the severe acute respiratory syndrome outbreak in Hong Kong. Emerg Infect Dis 2003;9:1042–5. 10.3201/eid0909.030366 14519237 PMC3016765

[19] Ajisegiri WS, Chughtai AA, MacIntyre CR A risk analysis approach to prioritizing epidemics: Ebola virus disease in West Africa as a case study. Risk Anal 2018;38:429–41. 10.1111/risa.12876 28810081 PMC5949606

[20] Adlam B Risk assessment tool (DAISY) for emerging human infectious diseases In: Health analysis & information for action (HAIFA), 2012.

[21] Bakar AA, Kefli Z Predictive models for dengue outbreak using multiple rulebase classifiers In: Electrical engineering and informatics (ICEEI), 2011.

[22] Bruce Aylward R, Sutter RW, Cochi SL, et al Risk management in a polio-free world. Risk Anal 2006;26:1441–8. 10.1111/j.1539-6924.2006.00840.x 17184391

[23] Kriss JL, Stanescu A, Pistol A, et al The world health organization measles programmatic risk assessment tool-romania, 2015. Risk Anal 2017;37:1096–107. 10.1111/risa.12669 27439071 PMC9245689

[24] Fraser C, Riley S, Anderson RM, et al Factors that make an infectious disease outbreak controllable. Proc Natl Acad Sci U S A 2004;101:6146–51. 10.1073/pnas.0307506101 15071187 PMC395937

[25] Decker RJ Homeland security: key elements of a risk management approach. Washington DC: General Accounting Office, 2001.

[26] Health NIo NIH guidelines for research involving recombinant DNA molecules (NIH guidelines), 2002.

[27] Taylor LH, Latham SM, Woolhouse ME Risk factors for human disease emergence. Philos Trans R Soc Lond B Biol Sci 2001;356:983–9. 10.1098/rstb.2001.0888 11516376 PMC1088493

[28] Coker RJ, Hunter BM, Rudge JW, et al Emerging infectious diseases in Southeast Asia: regional challenges to control. Lancet 2011;377:599–609. 10.1016/S0140-6736(10)62004-1 21269678 PMC7159088

[29] Sharara SL, Kanj SS War and infectious diseases: challenges of the Syrian civil war. PLoS Pathog 2014;10:e1004438. 10.1371/journal.ppat.1004438 25393545 PMC4231133

[30] Kruk ME, Freedman LP, Anglin GA, et al Rebuilding health systems to improve health and promote statebuilding in post-conflict countries: a theoretical framework and research agenda. Soc Sci Med 2010;70:89–97. 10.1016/j.socscimed.2009.09.042 19850390

[31] Charette M, Berrang-Ford L, Llanos-Cuentas EA, et al What caused the 2012 dengue outbreak in Pucallpa, Peru? a socio-ecological autopsy. Soc Sci Med 2017;174:122–32. 10.1016/j.socscimed.2016.12.010 28024241

[32] Guagliardo SA, Barboza JL, Morrison AC, et al Patterns of geographic expansion of aedes aegypti in the Peruvian Amazon. PLoS Negl Trop Dis 2014;8:e3033. 10.1371/journal.pntd.0003033 25101786 PMC4125293

[33] Cancedda C, Davis SM, Dierberg KL, et al Strengthening health systems while responding to a health crisis: lessons learned by a nongovernmental organization during the Ebola virus disease epidemic in Sierra Leone. J Infect Dis 2016;214:S153–63. 10.1093/infdis/jiw345 27688219 PMC5050485

[34] Müller J, Kretzschmar M, Dietz K Contact tracing in stochastic and deterministic epidemic models. Math Biosci 2000;164:39–64. 10.1016/S0025-5564(99)00061-9 10704637

[35] Seidel J Disease X: World health organisation issues global alert for potential pandemic, 2018.

[36] Heymann DL Control of communicable diseases manual : an official report of the American Public Health Association. communicable diseases manual. 19 edn Washington, DC: American Public Health Association, 2008.

[37] Schlegel M, Osterwalder JJ, Galeazzi RL, et al Comparative efficacy of three mumps vaccines during disease outbreak in eastern Switzerland: cohort study. BMJ 1999;319:352. 10.1136/bmj.319.7206.352 10435956 PMC32261

[38] Harper SA, Bradley JS, Englund JA, et al Seasonal influenza in adults and children--diagnosis, treatment, chemoprophylaxis, and institutional outbreak management: clinical practice guidelines of the infectious diseases society of America. Clin Infect Dis 2009;48:1003–32. 10.1086/598513 19281331 PMC7107965

[39] International Society for Infectious Diseases About ProMED-mail, 2010.

[40] HealthMap About HealthMap: Boston Children’s Hospital, 2015.

[41] School of Public Health and Community Medicine UNSW Sydney Epi-watch, 2016.

[42] Jones-Engel L, Engel GA Disease risk analysis: a paradigm for using health-based data to inform primate conservation and public health. Am J Primatol 2006;68:851–4. 10.1002/ajp.20292 16900505

[43] Frieden TR, Damon I, Bell BP, et al Ebola 2014--new challenges, new global response and responsibility. N Engl J Med 2014;371:1177–80. 10.1056/NEJMp1409903 25140858

[44] Li Q, Guan X, Wu P, et al Early transmission dynamics in Wuhan, China, of novel coronavirus-infected pneumonia. N Engl J Med 2020;382:1199–207. 10.1056/NEJMoa2001316 31995857 PMC7121484

[45] He X, Lau EHY, Wu P, et al Temporal dynamics in viral shedding and transmissibility of COVID-19. Nat Med 2020;26:672–5. 10.1038/s41591-020-0869-5 32296168

[46] Dato V, Wagner MM, Fapohunda A How outbreaks of infectious disease are detected: a review of surveillance systems and outbreaks. Public Health Rep 2004;119:464–71. 10.1016/j.phr.2004.07.003 15313109 PMC1497658

[47] Haydon DT, Cleaveland S, Taylor LH, et al Identifying reservoirs of infection: a conceptual and practical challenge. Emerg Infect Dis 2002;8:1468–73. 10.3201/eid0812.010317 12498665 PMC2738515

[48] Mitsakakis N, Wijeysundera HC, Krahn M Beyond case fatality rate: using potential impact fraction to estimate the effect of increasing treatment uptake on mortality. BMC Med Res Methodol 2013;13:109. 10.1186/1471-2288-13-109 24006924 PMC3847357

[49] Lago-Peñas S, Cantarero-Prieto D, Blázquez-Fernández C On the relationship between GDP and health care expenditure: a new look. Econ Model 2013;32:124–9. 10.1016/j.econmod.2013.01.021

[50] Gerdtham U-G, Jönsson B International comparisons of health care expenditure — conversion factor instability, heteroscedasticity, outliers and robust estimators. J Health Econ 1992;11:189–97. 10.1016/0167-6296(92)90035-Y

[51] Index GP Measuring peace in a complex world. Sydney: Institute for Economics and Peace, 2017.

[52] Alexander KA, Sanderson CE, Marathe M, et al What factors might have led to the emergence of Ebola in West Africa? PLoS Negl Trop Dis 2015;9:e0003652. 10.1371/journal.pntd.0003652 26042592 PMC4456362

[53] The Global Economy Economic indicators for over 200 countries, 2016.

[54] World Health Organization Health workforce requirements for universal health coverage and the sustainable development goals (human resources for health observer), 2016.

[55] New South Wales Department of Health Hepatitis A linked to imported frozen pomegranate, 2018 Available: https://www.health.nsw.gov.au/Infectious/alerts/Pages/hep-A-pomegranate.aspx [Accessed 15 Oct 2018].

[56] World Health Organization Measles – Japan. emergencies preparedness, response, 2018 Available: http://www.who.int/csr/don/20-june-2018-measles-japan/en/

[57] Scavia G, Alfonsi V, Taffon S, et al A large prolonged outbreak of hepatitis A associated with consumption of frozen berries, Italy, 2013-14. J Med Microbiol 2017;66:342–9. 10.1099/jmm.0.000433 28086079

[58] Blinder A Atlanta hospital admits second American with Ebola., 2014 Available: http://www.nytimes.com/2014/08/06/us/nancy-writebol-kent-brantly-ebola-atlanta.html?_r=0 [Accessed 22 Sept 2018].

[59] Heukelbach J, Alencar CH, Kelvin AA, et al Zika virus outbreak in Brazil. J Infect Dev Ctries 2016;10:116–20. 10.3855/jidc.8217 26927450

[60] ReliefWeb Bangladesh: diphtheria outbreak, 2017.

[61] World Health Organization Ebola response roadmap situation report, 2014.

[62] Nigeria Centre for Disease Control An update of Lassa fever outbreak in Nigeria, 2018.

